# Metabolomic Profiling of Commercial Tomato Puree by One-Shot Mass Spectrometry-Based Analysis: A Qualitative Perspective

**DOI:** 10.3390/metabo15110732

**Published:** 2025-11-09

**Authors:** Antonella Lamonaca, Elisabetta De Angelis, Rosa Pilolli

**Affiliations:** Institute of Sciences of Food Production, National Research Council of Italy, Via Amendola 122/O, 70126 Bari, Italy; antonellalamonaca@cnr.it (A.L.); elisabetta.deangelis@cnr.it (E.D.A.)

**Keywords:** tomato, single-shot extraction, metabolite profiling, untargeted metabolomics, high resolution mass spectrometry

## Abstract

Tomato is one of the most important vegetable crops worldwide, with about one quarter of the yearly production of fresh fruits dispatched to the processing industry. Paste, canned tomatoes, and sauces represent the three leading categories. **Background/Objectives**: The metabolic profile of processed tomatoes can be modified by several production steps, affecting the nutritional and sensory profile of the finished product. Despite this, a detailed metabolomic profiling of transformed tomatoes is currently missing. The goal of this investigation is to provide qualitative metabolomic profiling of tomato purees with two main advances: first, the use of a more sustainable analytical approach based on a single extraction protocol and one-shot analysis for multiple information retrieval on different compound classes; second, the achievement of a curated database consolidated over a wide collection of commercial samples representative of the Italian market. **Methods**: A non-selective ethanol extraction was applied to collect the main polar metabolites followed by untargeted high-resolution MS/MS analysis and software-based compound identification. **Results**: A list of more than five hundred features was collected and assigned to specific compounds or compound groups with different confidence levels. The results confirmed the persistence in processed tomatoes of the main primary and secondary metabolites already reported in fresh fruits, such as essential amino acids, sugar, organic acids, vitamins, fatty acyls, and phytohormones. Moreover, new insight on specific components never traced before in similar finished samples is provided. Bioactive compounds were detected in all samples, such as oligopeptides with ACE-inhibitor activity, ɣ-aminobutyric acid, alkaloids, and polyphenols (flavonoids, coumarins, and cinnamic acids). Many of these compounds have antioxidant activities, proving the relevance of transformed tomatoes as a source of health-promoting compounds for the human diet. **Conclusions**: A detailed metabolic profile of commercial tomato puree samples was obtained, and a curated database of metabolites was compiled, which can be useful for multiple purposes, for example, authentication, quality, or nutritional assessments.

## 1. Introduction

Tomato (*Solanum lycopersicum* L.) is the most important vegetable crop, with approximately 192.3 million tons of tomato fruits produced in 5.41 million ha each year. Asia accounts for 62.3% of global tomato production, followed by America, Africa, and Europe [1]. The prominent players in EU tomato production include Italy, Spain, Poland, Greece, France, and the Netherlands [2].

The tomato market is constantly growing, and it is estimated that it will increase from USD 156.86 billion in 2024 to USD 166.09 billion in 2025 at a compound annual growth rate (CAGR) of 5.9%. Such growth can be attributed to consumer demand and dietary trends, supply chain dynamics, government policies and regulations, climate, and environmental factors [3].

Beyond its economic relevance, tomato consumption is widely supported by its nutritional value. The popularity of this fruit is largely due to the health-promoting compounds it contains [4]. As such, it is considered a healthy food and a key component in the “Mediterranean diet” and in any balanced diet as well.

Tomatoes represent a good source of folate, vitamins A and C, carotenoids, phenolic compounds, and potassium [5]. Lycopene is the major carotenoid compound responsible for the characteristic deep red colour and has proved to bring beneficial effects on human health [6,7]. ß-carotene, which accounts for 1–5% of the total carotenoid content [8], represents the primary source of pro-vitamin A for humans [9]. ß-carotene also exhibits anticarcinogenic, gene-modulating, and free radical scavenging properties [10]. The deficiency of ß-carotene in the diet is correlated to conditions like blindness, dry eyes, and premature infant death [11]. Ascorbic acid (Vitamin C), tocopherol (vitamin E), and bioactive phenolic compounds (quercetin, kaempferol, rutin, naringenin, and lutein, as well as caffeic, ferulic, and chlorogenic acids) contribute to making tomatoes a fruit with unique beneficial properties on account of significant antioxidant characteristics [12]. However, only a few of them have already been assessed to be persistent in processed tomato products [13].

The regular consumption of tomatoes can combine a constant intake of multiple healthy nutrients, with an overall positive impact on numerous pathological and/or physiological states, including cancer, diabetes, and eye disease [14]. However, most of such beneficial effects have been proven to be related to fresh fruit consumption, whereas a large variety of tomato-derived commodities belong to the daily human diet.

On a global scale, about a quarter of the annual production of fresh tomatoes is dispatched to the processing industry, and this makes tomato the world’s leading vegetable for processing [15]. The three main producers of tomatoes for processing in 2024 were China, California, and Italy, respectively [15]. An increasing global consumption of processed tomatoes has been observed over the past decade, rising from approximately 30 million tonnes of fresh material equivalent in 2010/2011 to 37 million tonnes in 2022/2023. North America and the Western European Union are among the main consumer regions [15].

Tomato concentrates can be commercialized with several formulations, such as tomato juice, paste, puree, and sauce, differing in the processing degree, the allowed ingredients, and the percentage of water and tomato soluble solids [16,17].

Although the nutritional value of fresh tomatoes has been readily assessed [18,19], limited knowledge is currently available about finished products due to the diverse processing steps applied to fresh fruit during the industrial processes. Several steps—including washing, breaking, heating, the separation of seeds and peels, evaporation, filling, pasteurization, and storage—are typically involved in the production pipeline of tomato concentrates, and each may significantly affect the formulation of the final product [20].

A clear and complete understanding of the persistence of health-related compounds after industrial-scale tomato processing is currently missing. Only a limited number of investigations have been published, mainly focused on specific compounds or restricted classes of molecules. Interestingly, a review paper from 2022 tried to address whether industrially processed tomatoes were nutritionally comparable to fresh fruits; however, only twelve original research papers were found on this topic, and no definitive conclusion was drawn due to scarce data, inconsistent outcomes, and non-comparable results [8].

Some antioxidant molecules were prioritized in the past literature to trace the effects of specific processing steps on the nutritional value of final products. However, multiple trends were reported depending on the processing method and conditions (time, temperature, peeling method, etc.), the cultivars, the grown conditions, and finally, the reporting units, which were often not properly scaled to the dry weight of the relevant tomato product [8]. For example, the technological processing of fresh tomatoes into paste was found to differently affect the content and/or bioaccessibility of lycopene. Some studies reported an increase [21], others, a reduction [22], and others, no significant change [23]. Abushita et al. [21] reported that the content of ascorbic acid and tocopherols decreased up to 38% and 20%, respectively, along the processing steps to produce pasteurized paste, likely due to thermal effects. In contrast, carotenoids were found to be generally stable during heating, except for β-carotene, which decreased upon thermal treatment. Interestingly, lycopene provided less susceptibility to degradation than β-carotene due to the cis-isomerization of its chemical structure [21].

Provisional data were also reported on how flavonoids and other phenolic compounds were affected by the industrial processing steps and assayed both as total polyphenol (TPC) and total flavonoid content (TFC) [24,25,26] and as individual compounds (rutin [23], naringenin chalcone, and naringenin [27]). The changes in TPC, TFC, and individual phenolic compounds during industrial processing were different in different studies, as properly reviewed by Wu et al. 2022 [8]. Such inconsistent data were tentatively ascribed to two main issues. First, both the TPC and TFC were assessed with nonspecific colorimetric assays, whose results were affected by a sample matrix and interfering components. Second, the outcomes were likely to be the net effects of increased extractability due to the rupture of the cell structure during thermal processing and a compound loss due to thermal and mechanical processing. The balance between these opposite effects depended upon specific cultivars and processing conditions, thus affecting the conclusions of each investigation [8].

According to this scientific background, the current knowledge about the nutritional profile of industrial processed tomatoes is still limited and controversial because multiple factors and methodological restrictions impaired a complete and effective topic elucidation. The tomato cultivars, the growing conditions, the processing procedures, and the analytical methods (including sample preparation, analysis, and reporting units) together affected the observed trends for the main compound classes already investigated, namely carotenoids and polyphenols.

The aim of the present work is to provide a general metabolomic profiling of industrial tomato purees derived from a wide collection of commercial samples, representative of the Italian market. The collection included 43 samples encompassing different brands, batches, varieties, and geographical origins to overcome the limitations of previous focused studies and to provide a wide consolidated qualitative perspective. We selected a single non-selective extraction protocol based on ethanol to mainly characterize the polar metabolite fraction with a rationale bias for green solutions [28]. Carotenoids, which require apolar extraction conditions [19], were intentionally excluded from the investigation due to the selected extraction method, which was designed to prioritize polar metabolites. We performed an untargeted high-resolution mass spectrometry (HR-MS)-based analysis followed by software-based identification. The use of HR-MS provided a significant methodological advance in comparison to colorimetric assays in terms of susceptibility to matrix-related interferences. We analyzed the obtained pool of data to produce a curated database of metabolites representative of commercial tomato puree, with a focus on the compound classes boasting physiological relevance (amino acids, nitrogen bases, phytohormones, etc.) or beneficial health properties (bioactive peptides, alkaloids, polyphenol, etc.).

To the best of our knowledge, this is the first metabolomic study carried out on a wide representative collection of tomato puree commercial samples with two main advances. First, we applied a sustainable analytical approach in which a single extraction protocol (with a green solvent) was used to obtain multiple qualitative pieces of information of different beneficial compound classes; second, we created the first curated metabolite database, representative of the Italian industrial tomato purees, which can provide an added value for multiple purposes, including further metabolomic insights, authentication studies, and quality or nutritional assessments.

## 2. Materials and Methods

### 2.1. Sample Collection

A collection of 43 commercial samples of tomato purees with different indications in terms of varieties and geographical origin was purchased from the local market. The collection included a total of 18 different brands with up to 4 lots for each brand (see Table 1). The samples were anonymized, randomized, and stored at room temperature before analyses.

### 2.2. Extraction Protocol

The purees were homogenized for 15 min in a shaker at 200 rpm. A 10 g aliquot of each sample was collected and extracted with 20 mL of absolute ethanol. The ethanol was chosen as a green alternative option [28] to the well-established methanol-based protocols [18,19,20]. The mixtures were shaken on a vortex for 1 min, sonicated in a water bath for 5 min and finally mixed on an orbital shaker at 220 rpm for 5 min, at room temperature. Afterwards, the samples were centrifuged for 3 min at 4000× *g*, and the supernatants were recovered and filtered through a 0.45 µm regenerated cellulose syringe filter before LC-MS analysis. Please consider that such protocol only allowed for the characterization of alcohol-soluble compounds and was not expected to be effective in extracting lycopene and other carotenoids due to solubility constraints [19].

### 2.3. LC-MS/MS Analysis

Metabolic profiling of tomato puree samples was performed by HPLC-MS/MS analysis on an Ultimate 3000 UHPLC system (Thermo Fisher Scientific, Bremen, Germany) coupled to a hybrid quadrupole-Orbitrap^TM^ mass spectrometer Q-Exactive Plus (Thermo Fisher Scientific, Bremen, Germany). The metabolites were separated using reversed-phase liquid chromatography based on an Acclaim^TM^ 120, C18 analytical column (3 µm, 120 Å, 2.1 × 150 mm, Thermo Fisher Scientific, Bremen, Germany) at a flow rate of 200 µL/min.

Two solvents were used for chromatographic separation based on gradient elution: water + 2% methanol + 0.1% formic acid + 5 mM ammonium formate (A) methanol + 2% water + 0.1% formic acid + 5 mM ammonium formate (B). An elution multistep gradient was applied as follows: 0–25 min, solvent B increased from 5% to 25%; 25–30 min, solvent B increased from 25% to 50%; 30–31 min, solvent B increased up to 90% and then kept constant for 10 min; 41–42, solvent B decreased down to 5% and kept constant for 10 min for column conditioning. Column temperature was maintained constant at 25 °C along the run and the volume injection was 10 µL.

Untargeted high-resolution MS/MS analysis was performed both in the positive and negative ion modes, with independent runs, by FullMS/Data-dependent (FullMS-dd2) mode set up as follows: Full-MS event: scan range 80–1200 *m*/*z*, microscan 1, resolution 70 k, AGC target 1 × 10^6^, maximum injection time 30 ms; dd-MS^2^ event: microscan 1, resolution 17.5 k, AGC target 1 × 10^5^, maximum injection time 60 ms, loop count 10, isolation window 2.0 *m*/*z*, stepped collision energy 25, 35, 55; dd settings: minimum AGC target 5 × 10^2^, intensity threshold 8.3 × 10^3^, charge exclusion 3–8, >8, exclude isotopes on, dynamic exclusion 20 s. The following HESI interface and ion optics parameters were set: sheath gas flow rate 25, auxiliary gas flow rate 15, spray voltage 3.4 kV, capillary temperature 320 °C e S-lens RF level 55.

### 2.4. Data Processing and Annotation

HR-MS and MS/MS raw spectra were processed by commercial software Compound Discoverer v.3.3 SP2 (Thermo Fisher Scientific, Bremen, Germany). Multiple injections of the extraction solvent (blank samples) were carried out to allow the subtraction of background ions from the final list. Specifically, the software automatically hides in the feature list all the compounds which were found with a ratio lower than 1.5 (sample: blank) in the blank sample. The main parameters set to align, detect and group the experimental compounds into a list of features were itemized in the following: maximum precursor ion deviation 10 ppm; maximum retention time shift 0.2 min; minimum peak intensity 300,000 a.u. (arbitrary units) and S/N ≥ 3.

Specific nodes for features identification by accurate matching of either precursor ions (ChemSpider, Mass List) or fragmentation patterns (mzCloud, mzVault), were included with multiple databases and libraries selected for compound matching. The accuracy for annotation was fixed at Δm ≤ 10 ppm for precursor ions and ≤10 ppm for specific fragments.

The Chemspider search was set with the following public online databases: Carotenoids Database, FDA, Food and Agriculture Organization of the United Nations, FooDB, Lipids MAPS, Peptides, Pesticide Common Names, Phenol-Explorer and Toxin, Toxin-Target Database. The Mass List and mz Vault searches were set up with built-in software databases and libraries.(Compound Discoverer v.3.3 SP2, Thermo Fisher Scientific, Bremen, Germany)

The software output was manually curated by the analyst based on specific criteria set out by the ‘Metabolomic Standard Initiative’ [29,30]. In particular, the features were screened in compliance with the standard criteria of “level IIa” (compound annotation by matching of the HR-MS/MS fragmentation patterns–minimum of two characteristic fragments) and “level III” (putatively compound annotation by accurate mass matching of the precursor ion). As for identification level III, the full match of the isotopic pattern (M, M + 1, and M + 2) of the precursor ion was assessed.

### 2.5. Multivariate Statistical Analysis

In addition to the identification process, the software mentioned in Section 2.3 (Compound Discoverer v.3.3 SP2,Thermo Fisher Scientific, Bremen, Germany) provided basic statistical tools. The chromatographic peak areas expressed in arbitrary units and reported as the sum of different adducts retrieved for the same compound were assumed to be abundance-related features. The method reproducibility was assessed with a nested design over four independent batches of the same tomato puree. Principal component analysis (PCA) was carried out on all annotated metabolites (scaled and centred abundances) to highlight sample trends. Heat maps on scaled abundances and hierarchical cluster analysis (HCA) based on Euclidean distance and average linkage were reported as well.

## 3. Results

The aim of this investigation was an unspecific characterization of the metabolic profiling of commercial tomato purees by a sustainable extraction protocol and untargeted high-resolution mass spectrometry (HR-MS)-based identification. The food commodities were purchased from the local market, including a wide selection of brands, varieties, and geographical origins, to have a representative collection of commercial products commonly available in Italy for the daily diet.

After homogenization, a reproducible and non-selective alcohol-based extraction protocol was applied to obtain a wide recovery of diverse compound classes. No specific purification step was included in the protocol to avoid loss of information about the metabolic profile. Ethanol-based extraction was preferred to the more common methanol option to reduce the environmental impact of the applied method of analysis [28,31].

**Figure 1:**
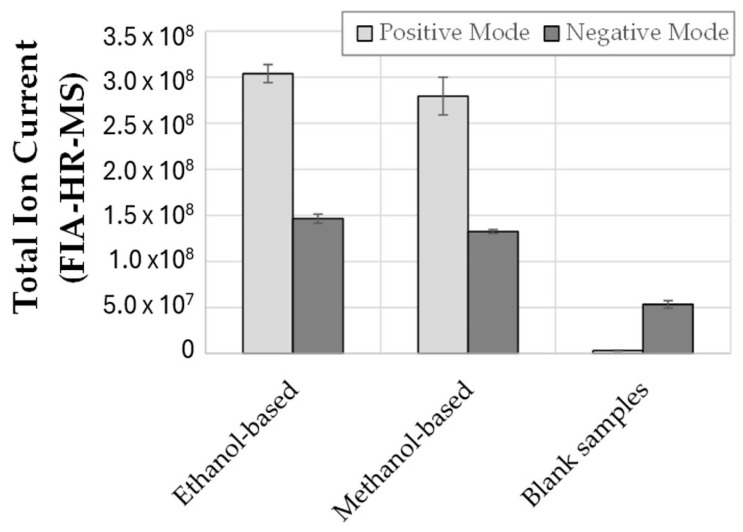
Extractability comparison between ethanol and methanol-based protocols (see Section 2.2 for the complete protocol). The total ion current (TIC) acquired upon flow injection analysis and high-resolution mass spectrometry acquisition (FIA-HR-MS) was presented in both positive and negative analysis modes. The experimental TIC values were averaged over three independent replicates, and the relevant standard deviation is displayed as an error bar.

A preliminary experiment was carried out to confirm comparable results between the two solvents in terms of overall extractability. Briefly, a set of test samples of tomato puree was alternatively extracted with methanol or ethanol under the same protocol (constant sample: solvent ratio, shaking, and ultrasound time) and analyzed by flow injection analysis and full MS acquisition (switching between positive and negative analysis mode). The total ion current (TIC) recorded for all samples was extracted and averaged over three independent replicates (see Figure 1).

No significant differences were assayed for the two solvents in positive acquisition mode, whereas a slight improvement in the overall signal (TIC) was recorded in negative acquisition mode compared to the methanol alternative option. Such experimental evidence confirmed what was already known from the literature about the viability of switching towards a more sustainable option in alcohol-based extraction protocols.

### 3.1. Metabolomic Data Acquisition, Processing, and Annotation

The whole collection of ethanol extracts was analyzed by reversed-phase HPLC and untargeted HR tandem mass spectrometry (HR-MS/MS) in both positive and negative polarity modes. The raw data were processed via software, Compound Discoverer v3.3 SP2 (Thermo Fisher Scientific, Bremen, Germany), to provide a tentative list of identification by matching precursor ion accurate mass and/or fragmentation patterns with deposited databases. Interestingly, the selected software allowed us to simultaneously manage both polarity acquisitions and to automate ions grouping (merge different adducts of the same compound), thus helping to speed up the unravelling of the complex metabolomic profile.

**Figure 2:**
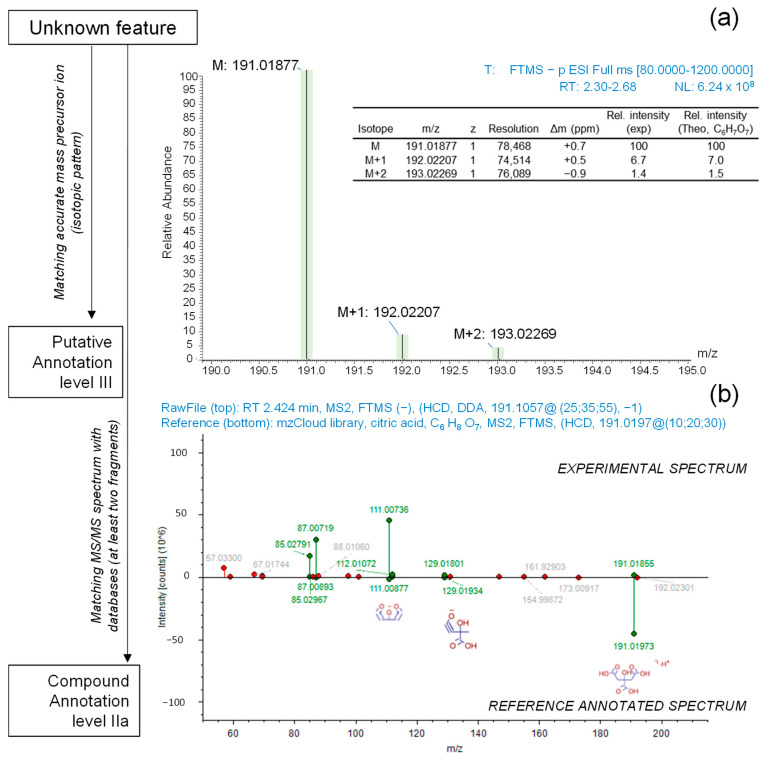
A typical example of the analytical workflow followed for each compound annotation at different identification levels. The reported MS spectra refer to the precursor ion 191.0188 *m*/*z* (panel **a**) and its fragmentation pattern (panel **b**) assigned to citric acid. Annotation level III was based on the accurate match of the isotopic pattern of the precursor ion (panel **a**); annotation level IIa was based on the accurate match of the fragmentation pattern (minimum of two specific fragments) with deposited databases (panel **b**).

The software output was manually curated to screen the most reliable annotation based on specific criteria set out by the ‘Metabolomic Standard Initiative’ [29,30]. Pursuing an untargeted approach without any aid of chemical standards, the highest level of identification accomplished here was level IIa, assigning compound annotation based on fragmentation pattern matching with a minimum of two characteristic fragments per compound. Secondary, “level III” identification was accomplished with putative compound annotation based on the accurate mass of the precursor ion and the full match of the isotopic pattern. In Figure 2, an example of the two levels of compound annotation was presented for citric acid: panel a reported the accurate matching of the precursor ion of the isotopic pattern, and panel b presented the annotated MS/MS fragmentation pattern matched with deposited databases.

The curated annotated list resulted in a total of 188 features assigned with level IIa, and 399 features putatively assigned with level III (see Table S2). Noteworthy, the list presented some examples of redundant identifications of different chromatographic peaks to the same compound. Such experimental evidence was ascribed to the presence of multiple isoforms that share the same molecular formula but differ in their structures or conformational arrangements, accounting for different elution times in reversed-phase chromatography. In the absence of chemical standards to support unambiguous peak picking, such redundant identification was maintained in the final list as output from the original software.

The annotated features were classified based on their chemical taxonomy retrieved by open-source databases [32] into ten main super classes: ‘organic acids and derivatives’, ‘organoheterocyclic compounds’, ‘lipids and lipid-like molecules’, ‘phenylpropanoids and polyketides’, ‘organic oxygen compounds’, ‘nucleosides, nucleotides, and analogues’, ‘benzenoids’, ‘alkaloids and derivatives’, ‘organic nitrogen compounds’, and ‘homogeneous non-metal compounds’ (Table 2). Depending on the structural complexity of the specific compounds, assignments to multiple classes among the aforementioned list can be envisaged for some features. Still, to avoid misleading information, each compound was uniquely classified in a single category and counted only once in Table 2.

### 3.2. Metabolite Profiling: Molecular Function and Beneficial Effects

#### 3.2.1. Organic Acids and Derivatives

The most populated class was represented by organic acids and derivatives. This class mainly includes carboxylic acids, amino acids, oligopeptides, and gamma-cheto acids, and derivatives of these compounds, some of which putatively originate from food contact materials.

Among the carboxylic acids, the presence of citric acid was confirmed as the most common natural acid in tomatoes. Previous MS-based investigations have already reported on this compound but only in fresh fruits (see Table 3). It is involved in the primary metabolism of fruit and has a strong influence on the sensorial qualities of the harvested product, accounting for the acid taste. In addition to its natural occurrence in fruits, citric acid is often artificially added as an ingredient to processed tomato commodities as food flavouring and preservative due to its antimicrobial activity. As for the amino acid (AA) class, several free amino acids have been detected, including essential (histidine, phenylalanine, methionine, arginine, tryptophan) and non-essential ones (glutamic and aspartic acids, asparagine, tyrosine, proline) (see Table 3). Noteworthy, some non-proteinaceous AAs and AA derivatives relevant for their role in the plant metabolic pathways or their bioactivity were detected as well. Among these, norleucine, ɣ-aminobutyric acid (GABA), stachydrine, n-(phenylacetyl) aspartic acid (PAA), and indoleacetylaspartate (IA-Asp) deserve to be mentioned, which were only investigated in fresh fruits (see Table 3).

Glutamic acid, aspartic acid, and GABA have been reported to be the most important free AAs in tomatoes from the quantitative point of view. The ɣ-aminobutyric acid (GABA) is a non-proteinogenic AA that has received much attention as a health-promoting functional compound. It was proven to provide important beneficial properties in reducing blood pressure in the human body [33]. It is also an important metabolite in plants and controls cytosolic pH under acid load via the GABA shunt pathway. Among the major crops, tomato is renowned for accumulating a relatively high level of GABA in its fruits, although the levels drastically change during fruit development: the GABA increases during the mature green stage and subsequently rapidly decreases during the ripening stage [34]. Most of the other free amino acids increased during ripening. Aromatic AAs were particularly relevant because they constitute precursors of the flavour volatiles produced by secondary metabolism pathways [33].

Stachydrine, also referred to as proline betaine, has been previously reported as a naturally occurring alkaloid in plants and bacteria; interestingly, it has been detected here for the first time in tomato samples. It has been widely studied for its potential health benefits and metabolic pathway [35].

The PAA and IA-Asp, conjugate of indol-3-acetic acid (IAA), belong to the class of auxins. Auxins are plant hormones which play a cardinal role in the coordination of many growth and behavioural processes in plant life cycles and are essential for plant body development. The IA-Asp, together with the indole-3-acetaldehyde, also detected in the annotation list, represent IAA metabolites. These latter ones were recognized to play a role in tomato growth and development, particularly in response to stress [36]. A complete elucidation of the physiological mechanism is missing [37].

Multiple features were assigned to oligopeptides, including the linear sequences from two to four amino acids, cyclic sequences of up to six amino acids, and depsipeptides. All the detected peptide sequences were subjected to in silico prediction of potential bioactivity [38,39]. The results are listed in Table S3; specifically, seven peptides detected in tomato puree samples raised our attention for potential functional properties (NILP, LVL, VF, IPI, LLL, LL, LF). The identified peptides might potentially exert mainly ACE-inhibitory activity [40] and dipeptyl peptidase IV (DPP-IV) inhibitory activity [41].

#### 3.2.2. Organoheterocyclic, Organic Oxygen Compounds and Alkaloids

Another widely populated class of compounds consists of organoheterocyclic compounds, which share the structural characteristics of aromatic cycles, including one or more nitrogen and/or oxygen heteroatoms. Among these, examples of nucleotide bases (purine and pyrimidines), quinolines, isoquinoline, pyridines, indoles, dihydrofurans, lactones, and benzopyrans were detected (see Table 3).

Adenine, guanine, cytosine, and uracil have been identified by matching the fragmentation pattern together with some derived nucleosides. These latter ones represent fundamental units of the genetic code and of the transcription process and are variously involved in the superpathways of arginine and polyamine biosynthesis, purine nucleotide salvage, indole-3-acetate conjugate biosynthesis, and jasmonoyl-amino acid conjugate biosynthesis.

Ascorbic acid (vitamin C), annotated here, was widely expected as an essential nutrient naturally present in fresh tomatoes and already reported in a previous MS-based study in processed derivatives [20]. It plays a key role in the tomato antioxidant potential [14].

In addition, nicotinic acid was identified among the heterocyclic compounds known for its important role in the primary and secondary metabolism of higher plants. Indeed, nicotinic acid, as a precursor of its amide derivative, constitutes the functional moiety of the pyridine nucleotide coenzymes, NAD and NADP, involved in energy metabolism and in many aspects of metabolic regulation [42]. In addition, nicotinic acid represents the building unit of many simple pyridine compounds, such as trigonelline, also included in the identified features and other more complex alkaloids resulting from secondary metabolism pathways.

**Table 3:** Short list of the annotated compounds detected and identified in this investigation (full list has been attached as Table S2 of the supplementary material). In the last column, a clear reference to a previous MS-based investigation reporting the same compound in tomato samples was reported, distinguishing between detection in either fresh fruits (*) or in processed products (**).

Chemical Taxonomy		Name	Formula	Δmass [ppm]	*m*/*z*	Reference Ion	Main Fragments	Ref.
*Class*	*Sub Class*							
Carboxylic acids and derivatives	Amino acids, peptides, and analogues	DL-Arginine	C_6_H_14_N_4_O_2_	−0.21	1751.189	[M + H] + 1	600.655; 700.659; 1160.709; 1580.926; 1751.191	[43] *
		DL-Glutamic acid	C_5_H_9_NO_4_	−0.37	1480.604	[M + H] + 1	560.504; 840.450; 1020.555; 1300.501; 1480.611	[19,43] *
		DL-Histidine	C_6_H_9_N_3_O_2_	−0.50	1560.767	[M + H] + 1	830.609; 930.452; 950.608; 1100.716; 1560.767	[43] *
		DL-Phenylalanine	C_9_H_11_NO_2_	−0.07	1660.863	[M + H] + 1	1030.546; 1070.496; 1200.810; 1310.494; 1660.865	[19,43] *
		L-(−)-Asparagine	C_4_H_8_N_2_O_3_	0.11	1330.608	[M + H] + 1	700.295; 740.244; 870.557; 880.400; 1160.351	[43] *
		DL-Tyrosine	C_9_H_11_NO_3_	0.11	1820.812	[M + H] + 1		[18,43] *
		L-Aspartic acid	C_4_H_7_NO_4_	−10.02	1320.288	[M−H]−1	880.389; 1140.182; 1150.023; 1320.289	[43] *
		Methionine S-oxide	C_5_H_11_NO_3_S	−0.58	1480.426	[M + H-H_2_O] + 1		
		L-Proline	C_5_H_9_NO_2_	2.02	1160.708	[M + H] + 1	700.669; 710.692; 1160.709	[43] *
		L-Norleucine	C_6_H_13_NO_2_	0.56	1321.020	[M + H] + 1	690.706; 860.970; 871.003	
		Stachydrine	C_7_H_13_NO_2_	−0.53	1441.018	[M + H] + 1	580.660; 840.814; 1441.018	
		γ-Aminobutyric acid	C_4_H_9_NO_2_	4.13	1040.710	[M + H] + 1		[19,43] *
		N-(Phenylacetyl)aspartic acid	C_12_H_13_NO_5_	−1.13	2500.718	[M−H]−1		
		Indoleacetylaspartate	C_14_H_14_N_2_O_5_	−0.30	2910.975	[M + H] + 1		
	Amino acids, peptides, and analogues	NILP	C_21_H_37_N_5_O_6_	−0.30	4562.812	[M + H] + 1	860.700; 1160.708; 2001.393; 2281.341; 3412.180	
		LVL	C_17_H_33_N_3_O_4_	−0.86	3442.540	[M + H] + 1	720.815; 860.970; 1321.020; 1851.650; 2311.702	
		VF	C_14_H_20_N_2_O_3_	−0.13	2651.546	[M + H] + 1	720.815; 2651.556	
		IPI	C_17_H_31_N_3_O_4_	−1.05	3422.384	[M + H] + 1		
		LLL	C_18_H_35_N_3_O_4_	−0.64	3582.696	[M + H] + 1		
		LL	C_12_H_24_N_2_O_3_	−0.80	2451.858	[M + H] + 1		[19] *
		LF	C_15_H_22_N_2_O_3_	−0.95	2791.700	[M + H] + 1		
	Tricarboxylic acids and derivatives	Citric acid	C_6_H_8_O_7_	−5.14	1910.187	[M−H]−1	850.297; 870.072; 1110.074; 1290.180; 1910.186	[19] *, [20] **, [43] *
Alkaloids	Not available	Trigonelline	C_7_H_7_NO_2_	0.05	1380.550	[M + H] + 1	920.500; 940.656; 1100.603; 1380.550; 1390.584	[43] *
Harmala alkaloids	Not available	_1_-methyl-_1_,_2_,_3_,_4_-tetrahydro-β-carboline-_3_-carboxylic acid	C_13_H_14_N_2_O_2_	−1.04	2311.126	[M + H] + 1		
Harmala alkaloids	Not available	Flazin	C_17_H_12_N_2_O_4_	−0.29	3090.869	[M + H] + 1		
Harmala alkaloids	Not available	Perlolyrine	C_16_H_12_N_2_O_2_	−0.48	2650.970	[M + H] + 1		[44] **
Phenols	Benzenediols	Kukoamine A	C_28_H_42_N_4_O_6_	−0.37	2661.624	[M + 2H] + 2		[45] *
Phenols	Methoxyphenols	Sinapyl alcohol	C_11_H_14_O_4_	−3.57	2090.812	[M−H]−1		
Benzene and substituted derivatives	Benzoic acids and derivatives	Salicylic acid	C_7_H_6_O_3_	−10.15	1370.230	[M−H]−1	930.330; 1370.231	[43] *
Fatty acyls	Fatty acyl glycosides	(−)-_11_-hydroxy-_9_,_10_-dihydrojasmonic acid _11_-β-D-glucoside	C_18_H_30_O_9_	−0.33	3891.820	[M−H]−1		
Fatty acyls	Fatty acyl glycosides	_12_-hydroxyjasmonic acid _12_-O-β-D-glucoside	C_19_H_30_O_8_	−0.79	3872.010	[M + H] + 1		
Fatty acyls	Linoleic acids and derivatives	(−)-trans-Methyl dihydrojasmonate	C_13_H_22_O_3_	−0.81	2271.640	[M + H] + 1		
Fatty acyls	Linoleic acids and derivatives	Methyl Jasmonate	C_13_H_20_O_3_	−0.51	2251.484	[M + H] + 1		
Prenol lipids	Sesquiterpenoids	(±)-(_2_E)-Abscisic acid	C_15_H_20_O_4_	−0.68	2651.433	[M + H] + 1		
Steroids and steroid derivatives	Steroidal glycosides	Tomatin	C_50_H_83_NO_21_	0.16	1034.516	[M + H] + 1		[19] *, [20] **, [46] **
Steroids and steroid derivatives	Steroidal alkaloids	Tomatidine	C_27_H_45_NO_2_	−1.04	4163.519	[M + H] + 1	1611.325; 2552.107; 2732.212; 3983.420; 4163.523	[43] *
Organooxygen compounds	Alcohols and polyols	D-(-)-Quinic acid	C_7_H_12_O_6_	−4.91	1910.552	[M−H]−1	850.279; 930.330; 1910.553; 1920.587	[18] *
Organooxygen compounds	Alcohols and polyols	Chlorogenic acid	C_16_H_18_O_9_	−0.12	3530.879	[M−H]−1		[19] *, [20] **, [43] *, [47] **, [48] **
Organooxygen compounds	Alcohols and polyols	Cynarine	C_25_H_24_O_12_	−0.32	5151.193	[M−H]−1		[48] **
Organooxygen compounds	Carbohydrates and carbohydrate conjugates	α-D-Mannose _1_-phosphate	C_6_H_13_O_9_P	−0.10	2590.224	[M−H]−1	789.575; 969.681; 1389.790; 1990.001; 2590.227	
Organooxygen compounds	Carbohydrates and carbohydrate conjugates	Sucrose	C_12_H_22_O_11_	−0.58	3411.088	[M−H]−1	690.343; 850.290; 1270.391; 1450.498; 1630.605	[19] *
Organooxygen compounds	Carbohydrates and carbohydrate conjugates	D-(+)-Glucose	C_6_H_12_O_6_	−4.77	1790.550	[M−H]−1		[19,43] *
Organooxygen compounds	Carbohydrates and carbohydrate conjugates	Glucosamine	C_6_H_13_NO_5_	0.21	1800.867	[M + H] + 1		
Organooxygen compounds	Carbohydrates and carbohydrate conjugates	D-Gluconic acid	C_6_H_12_O_7_	−4.84	1950.501	[M−H]−1		[19,43] *
Organooxygen compounds	Carbohydrates and carbohydrate conjugates	_1_-O-(_4_-coumaroyl)-β-D-glucose	C_15_H_18_O_8_	−0.27	3710.984	[M−H]−1		
Organooxygen compounds	Carbohydrates and carbohydrate conjugates	Caffeic acid _3_-glucoside	C_15_H_18_O_9_	−0.09	3410.878	[M−H]−1		[20,48] **
Organooxygen compounds	Carbohydrates and carbohydrate conjugates	Zeatin _7_-glucoside	C_16_H_23_N_5_O_6_	−3.65	3801.562	[M−H]−1		
Imidazopyrimidines	Purines and purine derivatives	Adenine	C_5_H_5_N_5_	−0.07	1360.618	[M + H] + 1	940.405; 1070.491; 1190.356; 1360.619; 1370.459	[43] *
Imidazopyrimidines	Purines and purine derivatives	Guanine	C_5_H_5_N_5_O	−0.35	1520.566	[M + H] + 1	1100.352; 1280.458; 1350.303; 1520.568; 1530.409	
Diazines	Pyrimidines and pyrimidine derivatives	Cytosine	C_4_H_5_N_3_O	2.47	1120.508	[M + H] + 1	690.455; 940.404; 950.245; 1120.509	
Diazines	Pyrimidines and pyrimidine derivatives	Uracil	C_4_H_4_N_2_O_2_	2.52	1130.348	[M + H] + 1	700.295; 850.289; 950.244; 960.085; 1130.349	
Pyridines and derivatives	Pyridinecarboxylic acids and derivatives	Nicotinic acid	C_6_H_5_NO_2_	0.77	1240.394	[M + H] + 1	780.344; 800.501; 960.448; 1240.395; 1250.429	
Indoles and derivatives	Indoles and derivatives	Indole-_3_-acetaldehyde	C_10_H_9_NO	−0.46	1600.756	[M + H] + 1		
Indoles and derivatives	Pyridoindoles	Norharman	C_11_H_8_N_2_	−0.14	1690.760	[M + H] + 1	1680.670; 1690.761	
Indoles and derivatives	Indolyl carboxylic acids and derivatives	DL-Tryptophan	C_11_H_12_N_2_O_2_	0.31	2050.972	[M + H] + 1	1180.655; 1440.809; 1460.601; 1590.919; 1880.707	[19,43] *
Dihydrofurans	Furanones	Ascorbic acid	C_6_H_8_O_6_	−6.50	1750.237	[M−H]−1	590.123; 710.122; 870.072; 1150.023; 1750.239	[20] **
Flavonoids	Flavans	(±)-Naringenin	C_15_H_12_O_5_	−0.67	2730.754	[M−H]−1	1070.124; 1190.489; 1510.026; 1770.184; 2710.616	[20] **, [19] *, [43] *, [46] **, [47] **, [48] **
Flavonoids	Flavans	Eriodictyol	C_15_H_12_O_6_	0.32	2870.562	[M−H]−1	830.123; 1070.124; 1250.230; 1350.440; 1510.026	[18] *, [43] *, [48] **
Flavonoids	Flavones	Quercetin	C_15_H_10_O_7_	−0.96	3030.496	[M + H] + 1	1530.183; 1770.548; 2290.495; 2850.402; 3030.501	[19] *, [43] *, [48] **
Flavonoids	Flavonoids	Apiorutin	C_32_H_38_O_20_	0.30	7411.891	[M−H]−1		[20] **
Flavonoids	O-methylated flavonoids	(±)-Hesperetin	C_16_H_14_O_6_	−0.36	3010.717	[M−H]−1		[48] **
Flavonoids	Flavonoid glycosides	Rutin	C_27_H_30_O_16_	1.02	6091.471	[M−H]−1	710.499; 850.290; 1290.548; 1530.183; 3030.501	[20] **, [43] *, [46] **, [47] **, [48] **
Flavonoids	Flavonoid glycosides	Astilbin	C_21_ H_22_ O_11_	0.14	4491.091	[M−H]−1		
Flavonoids	Flavonoid glycosides	isoquercetin	C_21_ H_20_ O_12_	−0.14	4651.026	[M + H] + 1		
Flavonoids	Flavonoid glycosides	Kaempferol _3_-O-glucoside-_7_-O-rhamnoside	C_27_H_30_O_15_	0.01	5931.515	[M−H]−1		[48] **
Flavonoids	Flavonoid glycosides	Naringin	C_27_H_32_O_14_	2.64	5791.735	[M−H]−1		[49] *
Flavonoids	Flavonoid glycosides	Prunin	C_21_H_22_O_10_	−0.15	4331.140	[M−H]−1		[43] *
Flavonoids	Flavonoid glycosides	Quercetin _3_- (_2_G-xylosylrutinoside)	C_32_ H_38_ O_20_	−2.05	7432.014	[M + H] + 1		[19] *
Coumarins and derivatives	Coumarins and derivatives	_7_-Hydroxycoumarine	C_9_H_6_O_3_	−0.41	1630.389	[M + H] + 1	790.549; 890.391; 1070.495; 1350.441; 1630.390	[43] *
Cinnamic acids and derivatives	Hydroxycinnamic acids and derivatives	Feruloylagmatine	C_15_H_22_N_4_O_3_	−0.46	3071.763	[M + H] + 1		
Cinnamic acids and derivatives	Hydroxycinnamic acids and derivatives	(E)-p-coumaric acid	C_9_H_8_O_3_	0.12	1650.546	[M + H] + 1		[18] *, [47] **, [48] **
Cinnamic acids and derivatives	Hydroxycinnamic acids and derivatives	N-Caffeoyltyramine	C_17_H_17_NO_4_	−0.78	2981.083	[M−H]−1		
Cinnamic acids and derivatives	Hydroxycinnamic acids and derivatives	N-trans-Feruloyloctopamine	C_18_H_19_NO_5_	−0.64	3281.190	[M−H]−1		
Cinnamic acids and derivatives	Hydroxycinnamic acids and derivatives	Paprazine	C_17_H_17_NO_3_	−0.51	2841.279	[M + H] + 1		

Among the alkaloid class, β-carbolines (βCs), naturally occurring in foods, plants, and biological fluids and tissues, have attracted much attention because they can exhibit an array of biological, pharmacological, and toxicological activities, acting on the central nervous system. These alkaloids are divided into tetrahydro-β-carbolines (THβCs) and aromatic βCs, whose occurrence in processed tomato products, fruit juices, and jams has already been reported in the literature [50].

The identification of compounds from both classes has been accomplished here; in particular, two features putatively assigned to isoforms of 1,2,3,4-tetrahydroharmane-3-carboxylic acid have been detected, and features assigned to norharman and harmala compounds (perlolyrine, flazine), belonging to aromatic βCs, have been putatively identified as well. Norharman is an indole alkaloid with reported antidepressant effects linked to neurotransmitter modulation [50]. Perlolyrine and flazin are both furans containing βCs, with bioactive properties [44,51,52].

Besides auxins, further (putatively) assigned compounds recognized as phytohormones for tomatoes, but structurally classified in other taxonomic groups, are zeatin, abscistic acid (ABA), jasmonates, and salicylic acid, all involved in multiple metabolic pathways related to fruit development and ripening [36].

The zeatin, detected here as a storage derivative form (zeatin glucoside), belongs to the cytokinin class involved in the early stages of fruit development just after pollination. Among the zeatin metabolites, the glucoside derivatives were reported to increase their content during ripening, suggesting a role in maintaining homeostasis of the bioactive hormone form (trans-zeatin) in tomato fruits [36].

ABA has been widely studied for its involvement together with ethylene in fruit ripening, but it is also involved in plant response to stress (abiotic stresses, such as drought and salt stress, and biotic stresses) [36,53]. In addition, it has been reported for its use as an exogeneous metabolite in post-harvesting practices [54].

Jasmonates and salicylic acid play roles in the regulation of fruit flavour aroma and shelf life. A jasmoic acid (JA) precursor (12-oxo phytodienoic acid) and four JA metabolites have been putatively identified as methyl jasmonate, methyl dihydrojasmonate, and two isoforms of 11-hydroxy-9,10-dihydrojasmonic acid 11-β-D-glucoside. The total amount of endogenous jasmonates is expected to fluctuate during fruit development and ripening [36].

Among the organooxygen compounds, some expected annotations of carbohydrates and alcohols deserve to be mentioned [55]. Glucose, sucrose, gluconic acid, glucosamine, and α-D-mannose 1-phosphate were identified. Gluconic acid has been naturally found in tomatoes, generated and accumulated in fruits during storage, likely due to the degradation of ascorbic acid: gluconic acid would be produced as an intermediate of the ascorbate production [56]. In addition, gluconic acid has been used as an additive in processed commodities for acidity regulation. α-D-Mannose 1-phosphate is an essential intermediate metabolite in the biosynthesis of mannosides in polysaccharides, glycoproteins, and glycolipids.

Quinic acid was also identified. The latter is a cyclic polyol with a carboxylic substituent, physiologically synthesized by a branch reaction of the shikimic acid pathway, and is considered as a reserve compound for phenolic biosynthesis. Numerous studies focus on the organic acids involved in the tricarboxylic acid cycle, but quinic acid was rarely analyzed in tomatoes. Recently, it has been reported that quinic acid, together with galacturonic, gluconic, and malonic acids, is associated with the softening and deterioration of cherry tomatoes during storage [56].

#### 3.2.3. Phenylpropanoids and Polyketides

Focusing on the secondary metabolism of tomato fruits, the phenylpropanoids and polyketides class play a crucial role. This class includes mostly flavonoids but also coumarins, cinnamic acids, and phenylpropanoic acids.

Flavonoids are predominant in fruits and vegetables, with specific physiological roles in plants but also important functional activity on human health upon regular intake through the diet [57].

Among the various types of flavonoids identified here (see Table 3), naringenin and naringin (glycoside derivative of naringenin) deserve to be mentioned for their antioxidant, anti-inflammatory, anti-ulcer, antiproliferative and antimutagenic properties [58,59]. Both compounds were already reported in tomato fresh fruit [19,43,49] and/or processed derivatives [20,46,47,48]. Quercetin, quercetin 3-(2G-xylosylrutinoside), rutin (quercetin 3-rutinoside), hesperetin, eriodictyol, apiorutin, and prunin were also identified, confirming previous MS data on their natural occurrence in tomato samples (see Table 3) [18,19,43]. Most of them, except for quercetin 3-(2G-xylosylrutinoside), were already assessed in tomato processed samples (sauce and paste) [20,46,47,48]. In addition, isoquercetin and astilbin were identified here for the first time in tomatoes. Most of these compounds may exhibit remarkable functional properties that will be discussed in the next section [60,61,62].

Coumarins, phenolic acids, and other secondary plant metabolites can provide various pharmacological activities. Umbelliferone (7-Hydroxycoumarin) has already been identified in tomato samples [43].

Chlorogenic acid, (E)-p-coumaric acid, caffeic acid 3-glucoside, cynarine, kukoamine A, feruloylagmatine, N-caffeoyltyramine, N-trans-feruloyloctopamine, paprazine, 1-O-(4-coumaroyl)-β-D-glucose, and sinapyl alcohol were the main identified compounds belonging to the class of cinnamic acids. Only a few of them were previously reported in tomato samples (see Table 3). The presence of chlorogenic acid in tomato fruit has been widely reported both in fresh fruits [19,20,43] and in finished products [47,48]. It is a phenolic compound formed by the esterification of caffeic acid with quinic acid and represents one of the most abundant polyphenols in tomatoes [63]. It can provide multiple benefits, being involved in the plant defence for its antioxidant properties and exerting health-promoting effects in humans upon regular intake by improving blood pressure, insulin sensitivity, and even promoting weight loss [64].

Kukoamine A is a polyamine alkaloid previously characterized in potato peels and in smaller quantities in tomatoes, peppers, and eggplant [45].

#### 3.2.4. Lipids and Lipid-like Molecules

The class of lipids and lipid-like molecules was also widely populated, including mainly fatty acyls, glycerophospholipids, and prenol lipids, steroids and steroid derivatives, sphingolipids, sesquiterpenoids, and glycerolipids. Although tomato fresh fruit and its processed derivatives contain very small amounts of lipids, identifying them is important for understanding their nutritional profile and potential health impact. Indeed, several studies have assessed the protective role of lipids on essential lipophilic molecules, primarily lycopene, limiting their thermal degradation and promoting their absorption [65].

Among fatty acyl subclasses, many compounds identified in this work are derivatives of linoleic acid. Linoleic acid (C18:2) is a key polyunsaturated fatty acid abundantly present in tomato seeds, playing an important role in both plant metabolism and human nutrition [66]. Other subclasses of compounds identified were fatty amides, fatty acid esters, and fatty acyl glycosides, with diverse roles in signalling, defence, and fruit flavour.

Glycerophospholipids are key components of plant cell membranes, and their presence in tomatoes is correlated to crucial roles in morphogenesis, metabolism, and response to environmental stress [67]. According to the literature, the main glycerophospholipids identified in tomato puree include glycerophosphocholines, glycerophosphoinositols, glycerophosphoethanolamines, glycerophosphates, and glycerophosphoserines [68]. These compounds vary in the length and degree of unsaturation of their acyl chains.

The relevance of tomato glycerophospholipids for human health has only been barely valued so far, probably due to their relatively low amount in tomatoes compared to other food sources (such as eggs, soy, or fish). Still, some evidence of bioactive properties ascribed to glycerophospholipid-derived molecules can be found in the literature [69].

Finally, the identification of tomatine and tomatidine in the tomato puree collection, both previously reported in tomato fruits, deserves to be mentioned [19,43]. Tomatine has been previously detected also in processed tomato samples [20,46]. Tomatidine is the aglycone steroid nucleus of α-tomatine; the latter is a tomato glycoalkaloid, present mainly in leaves and stems but also in fruits [70]. Tomatine plays fundamental defensive roles for the plant, such as antifungal and antimicrobial [71]. It is a bioactive molecule with very interesting pharmacological properties; at high doses, it can cause gastrointestinal disorders, whereas with a moderate intake, it boasts several beneficial effects [70].

### 3.3. Multivariate Statistical Analysis of Metabolite Profiles

Aiming at complementing the qualitative profiling of tomato purees, we conducted a preliminary quantitative study within the sample collection. We used the abundances of the identified compounds, derived from peak areas and expressed as arbitrary units, to provide a differential analysis (see Section 2.5 for details).

We assessed data reproducibility with a nested design over a sub-set of samples, namely four independent batches of the same tomato puree supply (same brand, variety, and geographical origin). The overall coefficient of variation (CV%), averaged on all the identified compounds, was lower than 25% in both positive and negative acquisition polarities. This result was very satisfactory because it also included production variability.

**Figure 3:**
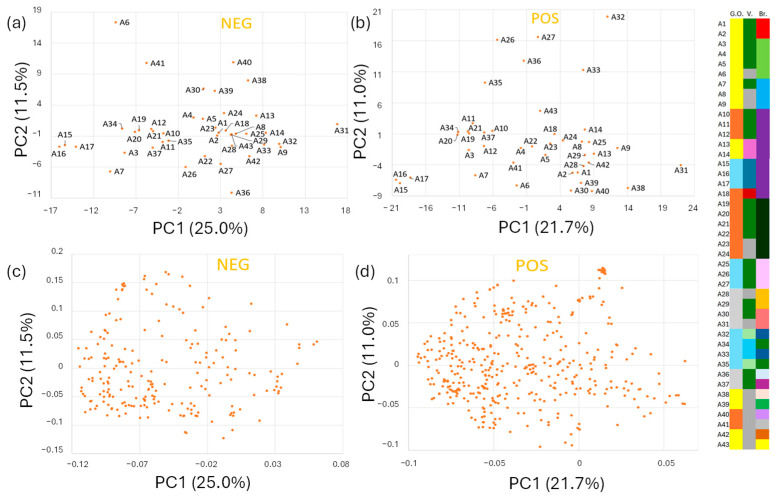
Principal component analysis (PCA) of the metabolites’ abundances, reported as arbitrary units from peak areas, obtained for all the annotated compounds. The colour coding on the right side of the figure describes the diversity of the sample collection with specific indications of geographical origin (G.O.), tomato variety (V.), and brand (Br.) of the samples labelled from A1 to A43. Panels (**a**,**b**) present the score plots of the PCA calculated on metabolites identified in negative and positive modes, respectively. Panels (**c**,**d**) present the loading plots of the PCA on metabolites identified in negative and positive modes, respectively.

We performed principal component analyses (PCA) on all annotated metabolite processing data from each polarity mode, separately. In Figure 3, panel (a) displays the score plot related to the metabolites detected in negative mode. Panel (b) reports the score plot of the positive mode. Relevant loading plots are represented in panels c and d, respectively.

The first two principal components retain only 35,5% and 31,7%, respectively, of the original variance. This confirms the complexity of the multivariate dataset, with low correlation among the variables. The orthogonal projection on the first two components discloses the intrinsic distribution of the commercial samples on account of the complex metabolite profile previously presented. Interesting trends can be valued. Most commercial samples with similar characteristics (geographical origin, variety, brand) hold similar coordinates in the score plots. See, for example, sample groups A1-A2, A10-A11-A12, and A15-A16-A17, to cite a few. In some cases, the similarity is confirmed beyond the commercial brand. See, for example, A1-A2 with A4-A5 and A3 with A7. However, clear discrimination between the sample groups on account of the different categorial factors cannot be achieved with this unsupervised statistical approach. Still, the consistency of these preliminary quantitative results represents a promising base for a detailed authentication study to be carried out by advanced statistical analyses.

**Figure 4:**
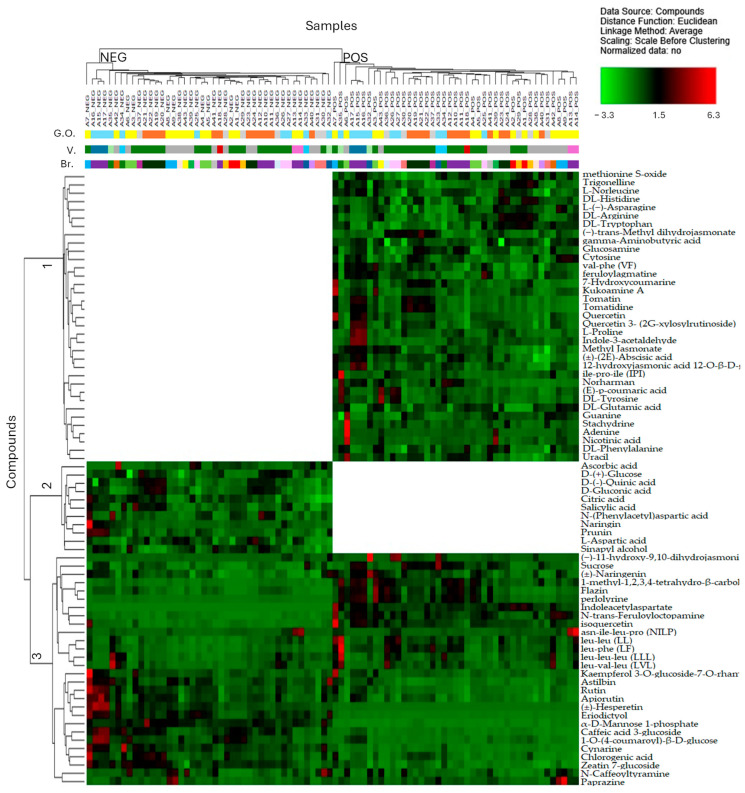
Hierarchical cluster analysis (HCA) and heat maps of the bioactive compounds. HCA was carried out with Euclidean distance function and average linkage method for abundance values, which were scaled before clustering. The colour coding on the top of the heat map describes the diversity of the sample collection with specific indications of the geographical origin (G.O.), tomato variety (V.), and brand (Br.) of the samples labelled from A1 to A43.

Finally, we accomplished a supplementary analysis of the differential distribution of the main bioactive compounds. We carried out the hierarchical cluster analysis (HCA) of abundance data to visualize similarity and grouping of specific commercial samples (see Figure 4). Abundances were scaled before clustering to avoid an overweight of the most intense peaks on the overall analysis. The heat map was used for visual clarity with a colour-coded HCA and dendrograms on the left and on the top of the heat map. These dendrograms displayed the cluster nodes of HCA. In particular, the dendrogram above the heat map disclosed the similarity among the tomato puree commercial samples. Two main clusters were obtained. These were the independent analyses of each sample in positive and negative modes (see Figure 4). Within each cluster, the samples with the same characteristics (geographical origin, variety, and brand) were progressively linked together, often with very small distances, as shown by the size of the dendrogram’s branch.

The dendrogram of the bioactive metabolites presented three main clusters. These consisted of the compound group detected only in positive mode (cluster 1), only in negative mode (cluster 2), and in both polarity modes (cluster 3). Interestingly, the HCA reported a consistent accumulation of polyphenols in samples A7, A15-A16-A17, made of baby plum and cherry tomatoes, respectively, with a clear geographical origin from Apulia and Sicily, respectively. In addition, the HCA disclosed a consistent accumulation of bioactive peptides in samples A35 (30% of cherry tomatoes from Sicily) and A13-14 (oblong tomatoes from Apulia).

## 4. Discussion

Tomatoes contain thousands of widely diverse metabolites that are broadly classified into primary metabolites, such as sugar, organic acid, fatty acids, nucleotides, and amino acids, and secondary metabolites, such as phenolic acids, flavonoids, and terpenoids. The natural content and the extractability of specific metabolite classes can be hugely different, and this poses an analytical challenge in accomplishing comprehensive metabolite profiling [18]. The global call for more sustainable behaviours and solutions also involved the academic research field, and considering this, in this investigation, we valued the chance to retrieve multiple pieces of metabolite information by one-shot analysis and green extraction protocols.

### Biological and Health Implications of the Main Identified Bioactive Compounds

The qualitative metabolite profile confirmed that a wide range of bioactive compounds previously reported only in fresh tomato fruit were also consistently detected in processed products. In addition, a short list of those compounds with health-promoting properties was identified for the first time in this investigation in tomato puree samples, providing new knowledge on this specific food formulation. The metabolite profile revealed a diverse spectrum of functional activities that collectively contribute to promoting the nutritional value of tomato puree, as an industrial formulation that typically belongs to the human daily diet.

Among the most relevant functional properties in tomatoes, antioxidant activity plays a crucial role, hindering oxidative stress and preventing cellular ageing. Vitamin C contributes significantly to the antioxidant properties of tomatoes, which can delay, inhibit, and prevent free radical oxidation by forming stable radicals [14]. In addition, a synergic contribution to this effect can be exerted by several identified compounds, including flavonoids, such as quercetin and its derivatives [60], naringenin and naringin [58,59], hesperetin [72], phenolic acids such as chlorogenic acid and p-coumaric acid [73], kukoamine A [74], and stachydrine [35]. The presence of all these antioxidants was proven in the industrial puree samples characterized in this work. Interesting anti-inflammatory activity is also well-supported by our data, accounted for by compounds such as stachydrine [35], quercetin and its glycosides [60], naringin and naringenin [58,59], p-coumaric acid [73], and tomatine [70], variously contributing to the modulation of inflammatory pathways. Moreover, glycerophospholipid-derived compounds were also found to modulate the inflammatory response [69].

Hypertension is a chronic medical condition that may affect human health as a trigger for potential cardiovascular complications. Therefore, the identification in tomato purees of several molecules that support cardiovascular protection, regulate blood pressure, and improve lipid profiles is equally relevant. Specifically, GABA is well-known for its hypotensive effects [34], whereas the ACE-inhibitory peptides (NILP, LVL, VF, LF), detected here for the first time in tomato puree, represent an important advance in the current literature because they can represent a natural alternative to synthetic hypertension drugs. Indeed, the use of synthetic ACE inhibitor drugs may pose significant adverse side effects such as dry cough, skin rashes, and headaches; on the contrary, recent studies reporting on the dietary intake of bioactive peptides did not indicate similar side effects in humans [40]. In addition to GABA and ACE-inhibitory peptides, chlorogenic acid [64], p-coumaric acid [73], and hesperetin [61] had beneficial effects on hypertension, preventing atherosclerosis, with a proactive effect in lipid metabolism. Finally, stachydrine was found to act as a naturally occurring vasodilator and anticoagulant [75].

Further health-promoting effects of tomato puree can be envisaged on glycemic control and metabolic regulation, supported by the presence of peptides with DPP-IV inhibitory activity (VF, IPI, LL), commonly used in type 2 diabetes management [41]. Quercetin [60,76], chlorogenic acid [64], and p-coumaric acid [73] can improve insulin sensitivity and contribute to glucose homeostasis. Glycerophospholipids also play a role in lipid metabolism and chronic disease prevention [69]. Noteworthy, a detailed study on flazine, identified in tomato juice, highlighted its potential in the treatment of diabetes by acting on insulin glycation/dimerization [52].

Neurological and psychotropic effects were attributed to norharman, the indole alkaloid detected in puree samples and associated with antidepressant activity via monoamine oxidase inhibition and neurotransmitter modulation [50], and neuroprotective properties were ascribed to kukoamine A [74].

Interestingly, potential anticancer and chemopreventive activities can be supported by a wide group of compounds. Prunin, which promoted apoptosis and inhibited cell proliferation [62], flavonoids such as quercetin [60] and naringenin and its glycoside [58,59], p-coumaric acid [73], coumarins [77], phenolic acids, and tomatine [70], all presented well-established antitumor effects. Less common compounds like perlolyrine and flazin, belonging to the β-carboline furan class, recently emerged as phase II enzyme inducers with promising chemopreventive potential [44,51]; however, only the limited and very recent literature is available about these compounds in foods [51].

Finally, several additional functional activities may contribute to the overall health-promoting potential of tomato purees. Vascular-related effects, including anticoagulant, anti-apoptotic, and angiogenic activities, were attributed to stachydrine [35,75]. Gastroprotective and anti-ulcer properties were exhibited by naringenin, naringin [58,59], and p-coumaric acid derivatives [73], all contributing to mucosal protection and ulcer prevention. Antimicrobial and antibiotic actions were demonstrated for quercetin and its glycosylated derivatives [60], umbelliferone [78], p-coumaric acid derivatives [73], and tomatine [70]. Tissue-protective effects, including skin regeneration, hepatoprotection, and renal support, were also ascribed to p-coumaric acid and its derivatives [73]. Immunomodulatory activity was described for tomatine, which enhanced the immune response and complemented its antimicrobial function [70].

In summary, the diversity of bioactive compounds identified in tomato puree confirmed its high relevance for the human diet not only as a valuable gastronomic component but also as a functional food with significant implications for preventive nutrition and health promotion.

## 5. Conclusions

In this investigation, we carried out an untargeted characterization of the metabolic profile of commercial samples of tomato puree by high-resolution mass spectrometry. The scientific goals were accomplished with a sustainable analytical approach based on green extraction protocols and high-throughput MS-based analysis. Noteworthy, the experimental results not only confirmed the persistence in processed tomatoes of well-established compounds previously reported in fresh fruit but also provided novel insights on specific components never traced before in similar finished samples, such as bioactive peptides. Moreover, some preliminary evidence of the potential accumulation of bioactive components in specific puree samples opens the way for new advances related to specific varieties and/or geographical origins.

The metabolite profile supported a diverse spectrum of functional activities which can promote the nutritional value of tomato puree as an industrial formulation. However, further quantitative analysis and in vitro assays should confirm proper concentration levels and preserved functional activity in the final ingested food. A perspective development of this investigation will focus on the missing class of carotenoids (e.g., lycopene), which are highly relevant for tomato quality and for its bioactive profile.

Finally, to the best of our knowledge, this study provides the first curated MS-based metabolite database, representative of the Italian industrial tomatoes purees, useful as a starting point for multiple applications and perspectives aimed at valorizing this product. For example, in the food industry, improved strategies tailored to preserve the stability of selected healthy tomato compounds upon processing might be set up, with consequent benefits for the final consumers. Furthermore, in the field of food authenticity, curated metabolomic profiles may represent the basic knowledge for the development of discriminant methods based on the detection of marker compounds.

In conclusion, our metabolomic results pave the way for multiple developments of the investigation into independent works, all confirming the high relevance of tomato puree for the human diet not only as a valuable gastronomic component but also as a functional food with health-promoting properties.

## Figures and Tables

**Table 1:** Description of the commercial sample collection (the full detailed list is reported as Table S1 and includes the sample coding).

Variable	Type	Counts/Type	Total
Variety	Baby Plum (Datterino)	20	43
	Oblong (Allungato)	2	
	Cherry (Ciliegino)	3	
	Rossoro	1	
	30% Baby Plum (Datterino)	2	
	30% Cherry (Ciliegino)	2	
	Not specified	13	
Geographical origin	Emilia Romagna	12	43
	Sicily	10	
	Apulia	15	
	Italy	6	
Farming condition	Organic	12	43
	Conventional	31	

**Table 2:** Chemical classification of the detected and annotated metabolites.

Super Class	N° Identified Features	
	Level IIa	Level III
Organic acids and derivatives	34	100
Organoheterocyclic compounds	19	30
Lipids and lipid-like molecules	14	103
Phenylpropanoids and polyketides	11	23
Organic oxygen compounds	4	35
Nucleosides, nucleotides, and analogues	6	5
Benzenoids	5	17
Alkaloids and derivatives	1	7
Organic nitrogen compounds	2	2
Homogeneous non-metal compounds	0	2
Other	4	74

## Data Availability

All data have been included in the main text and in the Supplementary Materials.

## Supplementary Materials

The following supporting information can be downloaded at https://www.mdpi.com/article/10.3390/metabo15110732/s1; Table S1: List of anonymized commercial samples of tomato puree; Table S2: Full list of annotated features. Table S3: Identified oligopeptides with putative bioactivity predicted in silico.

## Abbreviations

The following abbreviations are used in this manuscript:

MS	Mass Spectrometry
LC-QTOF-MS	Liquid Chromatography Quadrupole Time of Flight Mass Spectrometry
LC	Liquid Chromatography
UHPLC	Ultra-High-Pressure Liquid Chromatography
AGC	Automatic Gain Control
HESI	Heated Electrospray Ionization
HR-MS	High-Resolution Mass Spectrometry
S/N	Signal/Noise
IAA	Indol-3-acetic acid
DPP-IV	Dipeptyl peptidase IV
ACE	Angiotensin converting enzyme
AAs	Amino acids
GABA	ɣ-aminobutyric acid
PAA	n-(phenylacetyl)aspartic acid
IA-Asp	Indoleacetylaspartate
NAD	Nicotinamide adenine dinucleotide
NADP	Nicotinamide adenine dinucleotide phosphate
βCs	β-carbolines
THβCs	Tetrahydro-β-carbolines
ABA	Abscistic acid
JA	Jasmoic acid
